# Bioinspired skin towards next-generation rehabilitation medicine

**DOI:** 10.3389/fbioe.2023.1196174

**Published:** 2023-05-09

**Authors:** Zhenghui Wang, Chen Xiao, Mridul Roy, Zhiyao Yuan, Lingyu Zhao, Yanting Liu, Xuejun Guo, Ping Lu

**Affiliations:** ^1^ Department of Rehabilitation, The First Affiliated Hospital of Xinxiang Medical University, Weihui, China; ^2^ Department of Oncology, The First Affiliated Hospital of Xinxiang Medical University, Weihui, China; ^3^ SanQuan College of Xinxiang Medical University, Xinxiang, China

**Keywords:** bioinspired skin, rehabilitation medicine, electronic skin, tissue-engineered artificial, prostheses and implant

## Abstract

The rapid progress of interdisciplinary researches from materials science, biotechnologies, biomedical engineering, and medicine, have resulted in the emerging of bioinspired skins for various fantasticating applications. Bioinspired skin is highly promising in the application of rehabilitation medicine owing to their advantages, including personalization, excellent biocompatibility, multi-functionality, easy maintainability and wearability, and mass production. Therefore, this review presents the recent progress of bioinspired skin towards next-generation rehabilitation medicine. The classification is first briefly introduced. Then, various applications of bioinspired skins in the field of rehabilitation medicine at home and abroad are discussed in detail. Last, we provide the challenges we are facing now, and propose the next research directions.

## 1 Introduction

The skin is the body’s first line of defense that provides protection against harmful foreign substances and plays a key role in maintaining the body’s homeostasis (Li, 2017). As the largest organ in the body, it plays a wide range of functions, including respiratory, protective, secretory, thermoregulatory and sensory stimulation functions (Liang et al., 2022; Xie, 2022). Burns and ulcers cause about 9 million cases of skin lesions in China each year, of which 3.2 million require skin grafts. Additionally, more than 200,000 cases of burns require extensive skin replacement. Among them, some patients are unable to undergo autologous skin grafting due to having too many skin defects of their own. When allogeneic skin grafts, such as allografts and xenografts are chosen, the sources are extremely limited and there is a high probability of severe immune rejection of allogeneic skin (Zhao et al., 2017). For amputees, conventional prostheses only offer basic cosmetic and simple mobility functions, but they lack the tactile and sensory abilities that disabled patients really need. Therefore, the availability of high-quality skin remains a major factor limiting the development of rehabilitation medicine and affecting the health of patients. At this point, bioinspired skin with multiple functions is attracting significant interest as a novel area of research not only in human-machine interfaces, flexible wearable devices and soft robotics (Lei and Wu, 2018; Xing, 2021), but also has extraordinary significance for the development of rehabilitation medicine. Bioinspired skin technology has gained wider use in the field of rehabilitation medicine in recent years. For example, the development of skin tissue engineering has brought benefits to a large number of patients who need skin implants (Horch et al., 2005), while smart e-skin/bionic skin has made great progress in medical robotics and smart prosthetics (Wang et al., 2023). Bioinspired skin can improve the ability to transfer information between human-machine interfaces and increase the degree of human response to painful stimuli. Moreover, they are able to help patients regain self-care in daily life, regulate emotions and reconstruction to perform complex limb functions.

## 2 Classification of bioinspired skin

**FIGURE 1:**
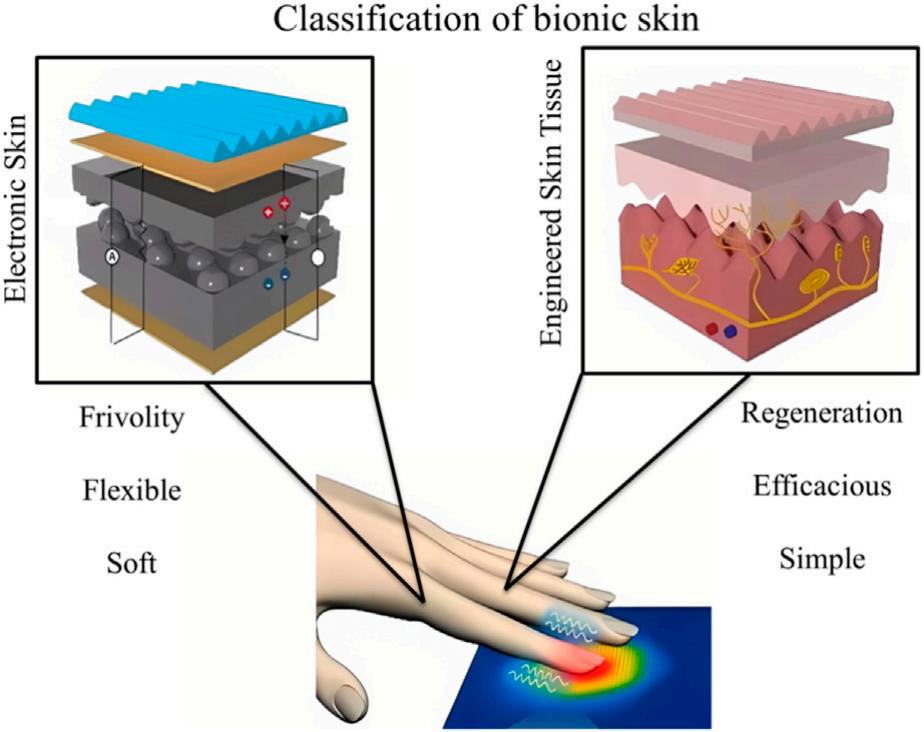
Classification of bionic skin: electronic skins and skin tissue engineering.

At this stage, research on bioinspired skin is mainly focused on two aspects: the first one is electronic skin (E-skin) and the second is tissue-engineered artificial skin (Figure 1). Early E-skin consists of rubber, conductive graphite and new transistors that can mimic the sensory functions of human skin (Lei, 2022). In recent years new materials such as polydimethylsiloxane, carbon nanotubes, graphene and hydrogels have been used to prepare E-skin sensors (Wan et al., 2020), making E-skin more convergent to human skin in terms of performance. Contrary, tissue-engineered artificial skin is being developed based on theories and methods from cell engineering and biology (Lin et al., 2022). And this skin substitute is artificially developed *in vitro* to repair and replace defective skin tissue (Naves et al., 2016).

### 2.1 Electronic skin

As the largest organ of the human body, the skin possesses a variety of interesting properties such as stretchability, self-healing ability, high malleability and haptic ability. Devices that mimic these properties of human skin, as well as additional functions, are referred to as electronic skins (Xiang, 2021). Advances in materials, mechanics, electronics and information technology have driven the development of smart electronic skins. For instance, due to the advancement of stretchable polymer materials, a wide variety of stretchable bioinspired sensors, such as pressure/strain sensors, thermal sensors, optical sensors and biochemical sensors, have been developed (Park et al., 2018). New organic semiconductors have extended the frontiers of smart electronic skin to integrate artificial nerves, including synapses and afferent nerves (Liu et al., 2022). Smart E-skins are exceedingly attractive in every respect. Although still under development, ionic skin, the next-generation of E-skin, is within reach of future development (Gong et al., 2022).

### 2.2 Tissue-engineered artificial skin

Skin tissue engineering is a modern approach to reconstruct structural and functional components of skin following chronic wound that generally occurs during the healing process and precludes skin regeneration. Currently, the scaffolding materials for tissue-engineered artificial skin are divided into two main categories: natural polymer materials and synthetic polymer materials (Wang et al., 2020). Tissue-engineered artificial skin can be broadly categorized into three types based on their structure and function: epidermal substitutes, dermal substitutes and skin substitutes containing a bilayer structure of epidermis and dermis (Yan et al., 2018). The development of 3D printing technology has significantly expanded the application of tissue-engineered skin in rehabilitation medicine owing to its excellent properties such as high resolution, flexibility, reproducibility and high throughput that paves the way for the preparation and medical application of tissue-engineered skin (Lian et al., 2021).

## 3 Distinctive features of electronic skin: stimuli-responsiveness and self-healing capacity

Wearable electronic skins can transform environmental stimulus variation into electronic signal change (Lee et al., 2020). To imitate this sensory ability of human skin, different sensitive conductive architectures are placed upon soft polymer substrates (Table 1). Guo et al. have provided a comprehensive review of the recent advances in the design strategy of material structures that imitate the multiple stimuli perception and self-healing functionalities of biological skins (Guo et al., 2022). The electronic sensors with the capacity to detect external stimulus include resistance-, capacity-, piezoelectric-, triboelectric, and potentiometric-type sensors (Yao et al., 2017). Through the unique coaxial structure and fibrous sensing architecture the sensor arrays could simultaneously map and quantify multiple mechanical stresses, including normal pressure, lateral strain and flexion. The capacitive sensor is one of the most traditional and popular sensors in tactile stimulation detection. Capacitance is a parameter to measure the charge storage capacity, and it is a function of the dielectric constant, area and the distance between the two electrodes of the parallel plate capacitor. The principle of the traditional capacitive sensor is that the pressure or shear force applied will lead to a change in the distance or area between the two plates, which will change the capacitance (Chou and Lee, 2021). Based on this principle, super-stretchable capacitive strain sensors and high-sensitivity capacitive pressure sensors based on various nanomaterials can be developed.

**TABLE 1:** Materials and working principles used for Sense and self-healing characteristics of bioinspired skin.

Stimuli	Material	Working principle
Force	MXene nanosheets, MNOH, PDMS, Single Crystalline Silicon (Kim et al., 2014; Liao et al., 2019; Zhang L et al., 2021; Chen et al., 2022; Cheng et al., 2022)	These materials can make electronic skin have tensile properties. The sensor has a variety of functions because of its special materials, conduction methods (piezoresistive, piezoelectric, capacitance, *etc.*) and special arrangement structure (serpentine, paper-cut, *etc.*). (Xu et al., 2013; Nasreldin et al., 2020)
Temperature	MoSe_2_, MoO_3_, Graphene, ZnO, nanometer material, CNTs (Kang et al., 2018; Liu et al., 2019; Zeng et al., 2022; Zhang H et al., 2022)	
Humidity		
Healing	MoS_2_、Silicon wafer、PDMS、3D porous graphene, Tattoo-base paper, Silicon/SU8, Single-walled carbon nanotube, PET film, Gold nanowires (García-Ávila et al., 2021; Wei et al., 2021; Cao and Cai, 2022; Li et al., 2022)	

Biological skins can recover their original appearance and critical functions after physical damage. Advanced e-skins are equipped with the self-healing ability to extend their service life and reduce maintenance costs (Keng et al., 2018). In order to achieve satisfying self-recovering properties, self-healable polymeric materials are specially designed and applied as the core functional component for self-healing electronics. According to different healing mechanisms, they could be generally categorized into extrinsic and intrinsic self-healing materials. Among them, single dynamic crosslinking networks are utilized to dynamically crosslink polymer materials and endow them with autonomous self-healing capabilities (Oh and Bao, 2019). Most of the organic polymer systems of flexible electronic skin materials are formed by the entanglement of long chains of polymer molecules, and when conditions such as temperature and humidity permit, the material itself can be reconstructed by the regeneration of dynamic covalent or non-covalent bonds and the re-entanglement of polymer chains at the damaged interface. Secondly, even if some polymers themselves cannot achieve self-healing, they can heal for a certain number of times after injury by adding a healing agent to the base material of the electronic skin in advance (Guo et al., 2022).

## 4 Application of bioinspired skin

### 4.1 Application of bioinspired skin in prostheses

**FIGURE 2:**
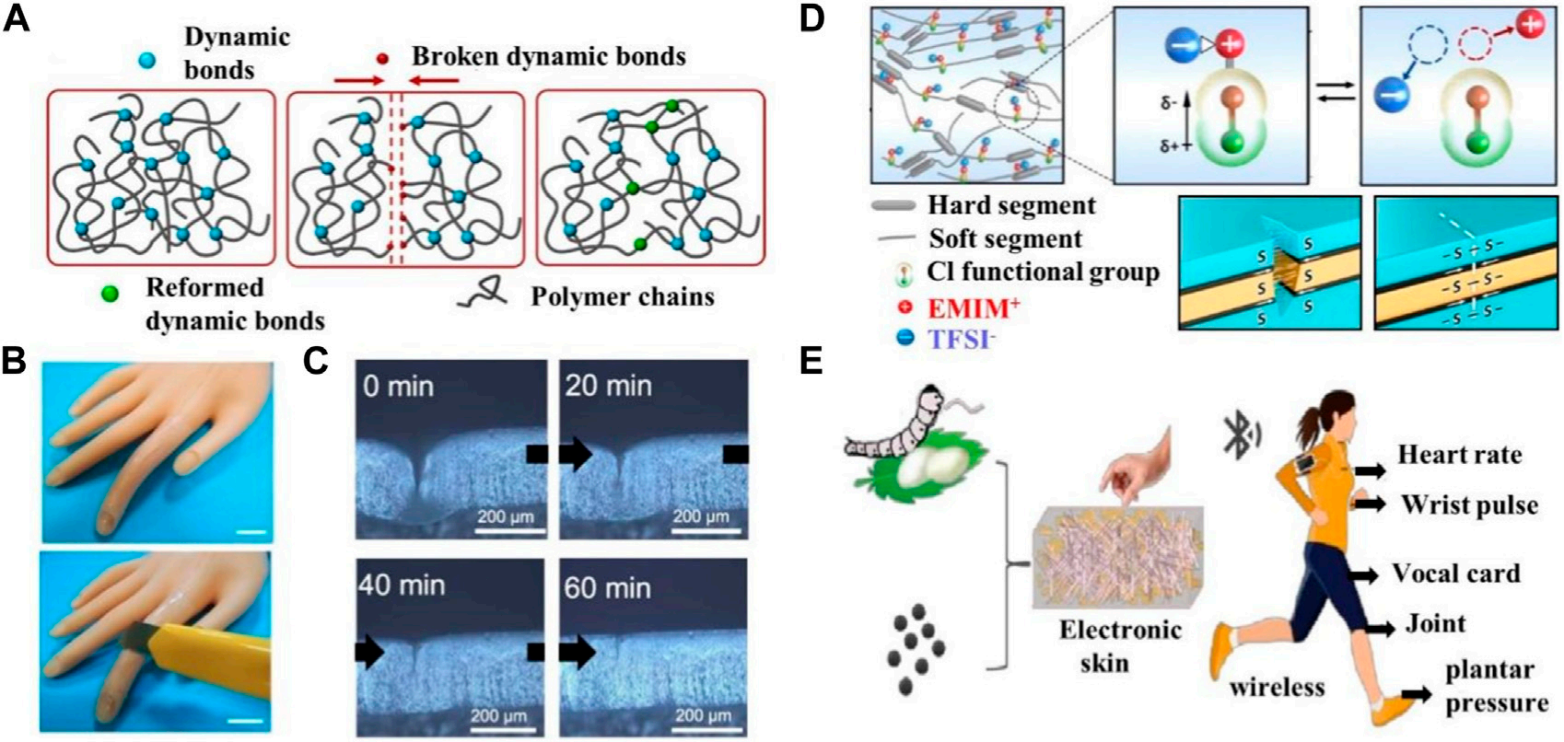
Application of bionic skin in prostheses. **(A)** The schematic illustration of the dynamic process of how dissociated groups tend to reform bridges to repair the networks for self-healing. Reproduced with permission from (Lei and Wu, 2018). **(B)** biomimetic skin has self-healability comparable to natural skin and can autonomously repair cracks. **(C)** Microscopic image observes the autonomous self-healing of a scar within 60 min at room temperature. **(D)** Representation of the conceptual design of the Cl-functionalized iontronic pressure-sensitive material (CLiPS) that uses dynamic disulfide bonds to construct the backbone for achieving autonomous self-healing properties to emulate the remarkable functionalities of human skin. Reproduced with permission from (Boahen et al., 2022). **(E)** Preparation process of SF/GO Nanofiber Composite flexible electronic skin. Reproduced with permission from (Jie, 2022).

The importance of a good pair of prostheses for disabled patients cannot be overstated, and the use of smart bioinspired skins with multiple functions in prostheses can make a world of difference to the lives of people with disabilities (Wang et al., 2022). Lei and Wu (2018) reported on a bioinspired smart skin based on supramolecular hydrogels. The transparent supramolecular hydrogel can polymerize into a series of skin-like or even skin-exceeding mechanical properties, combining compressive resilience, large stretching, self-healing, and can be shaped at will at room temperature by building multiple dynamic cross-linking networks with basic random copolymerization reactions (Figure 2A). When applied to the plastic fingers of a prosthesis, this supramolecular hydrogel-based bioinspired skin allows the prosthetic fingers to sense strain and temperature stimuli through capacitive and resistive signals, effectively mimicking the mechanical and temperature receptors of human skin. It can therefore record the bending and straightening movement of the finger based on the change in capacitance during the deformation process, and detects the rise in temperature of the prosthetic surface utilizing a real-time reduced resistance signal during manual contact with the prosthesis, allowing visual observation of the movement of the finger and the external temperature change (Figures 2B, C). However, as an exposed part of the prosthetic skin, it is susceptible to accidental mechanical damage caused by continuous wear and tear, resulting in interruption of function or reduced device life. Therefore, the ability to self-repair, similar to human skin, is an important inherent property necessary to restore damaged function to ensure stability and increase the longevity of the device. Boahen et al. (2022), designed and synthesized a novel thermoplastic polyurethane material containing dynamic disulfide bonding functional groups and chlorine substituents (Figure 2D). The material was based on the principle of mechanical stimulus-response of tactile cells, simulating the self-healing function of real human skin and biological ion signaling mechanisms. The dynamic disulfide bonding is always reversible and dynamic, enabling rapid self-healing of injuries at room temperature without additional energy. This reduces the frequency of prosthetic skin replacement, extends the life of the prosthesis, reduces the incidence of phantom limb pain and improves perception (Park et al., 2018). The new concept of ionic skin technology presented by this thermoplastic polyurethane material is of significant meaning as it simultaneously restores wound and haptic functions, making it an ideal candidate for future applications in human-computer interaction and wearable devices. Jie Zheng of the Zhejiang University of Technology (Jie, 2022) has developed a flexible electronic skin made of silk protein/graphene oxide (SP/GO) nanofibers (Figure 2E). This e-skin can be used to wirelessly monitor human joint movements and act as a pulse monitor to monitor human heart rate. The skin maintains stable sensing performance after 36,000 bend-release cycles, using a self-assembled wireless configuration to achieve SF/GO nanofiber composite flexible electronic skin. The SF/GO nanofiber composite flexible electronic skin can transmit data for real-time monitoring by connecting wirelessly to a smartphone. Park et al. have made great efforts in bioinspired skin electronic sensors which allow people with disabilities to touch the world with the help of haptic sensors (Park et al., 2018). Researchers at RMIT University in Australia (Rahman et al., 2020) have developed a new electronic skin by combining three technologies previously pioneered and patented by the team: stretchable electronics, self-modifying coatings, and electronic memory cells. The resulting skin can be used to improve prosthetic interfaces, increase grip accuracy and enhance the ability to identify the source of injury, allowing the prostheses to act like human skin. Being able to perceive various pain sensations, it acts and responds as quickly as human skin, transmitting pain as a neural signal to the human brain, enabling the prosthetic limb to feel pain like a normal limb that can help people with disabilities to rediscover danger.

### 4.2 Application of bioinspired skin in rehabilitation equipment

**FIGURE 3:**
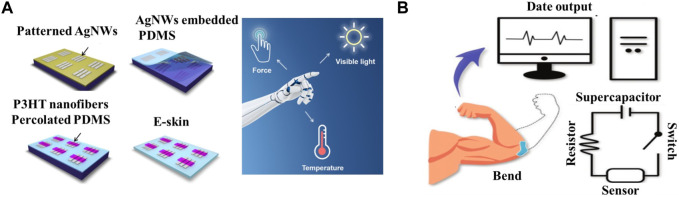
Application of bionic skin in rehabilitation equipment. **(A)** Schematic illustration of the process for fabricating the stretchable E-skin. Used with permission from (Liu et al., 2022). Sketch of the E-skin applied on an intelligent robot for multiple perceptions of force, temperature, and visible light. **(B)** Schematic illustration of the self-powered E-skin and corresponding sensing process. Reproduced with permission from (Liang et al., 2022).

Autonomous artificial intelligence rehabilitation robots with multi-sensory surfaces can perform rehabilitation diagnosis, while robots with sensory and sensing functions can perform highly interactive tasks such as rehabilitation therapy, and disease monitoring using bioinspired sensing skin applied on or in the body (Hammock et al., 2013; Yang et al., 2019). Professor Mao and the team at Zhengzhou University (Liu et al., 2022) have developed an electronic skin using P3HT nanofiber permeable PDMS composites as intrinsically stretchable polymer semiconductors. The e-skin is capable of multiple senses such as force, temperature and visible light (Figure 3A). Using this electronic skin, an intelligent robotic sensing and control system was further developed, that can recognize robotic hand gestures, measure the temperature of the touching object and control the robotic hand with visible light beams. The strategy of using intrinsically stretchable polymer semiconductors to construct multi-sensory electronic skins with simple structures is expected to accelerate the development of artificial robotic skins. The bioinspired skin is also expected to replace hospital monitoring instruments, enabling the measurement of vital signs such as heart rate and other health physiological signals to parse out the wearer’s health and physical condition and identify potential risk of disease onset promptly (Yao et al., 2020). A flexible electronics research team led by Professor Lan Wei (Liang et al., 2022) from Lanzhou University has proposed an all-in-one self-powered, fully transparent and flexible E-skin, which consists of a transparent supercapacitor, a stretchable transparent strain sensor and a serpentine resistor (Figure 3B). The flexible transparent supercapacitor was constituted using oxygen vacancy-rich molybdenum oxide nanowires as the active material and Nano-cellulose to modulate the refractive index of light to form a self-supporting paper electrode and it exhibited excellent flexibility, transparency and electrochemical performance. The “island bridge structure” strain sensor constructed from one-dimensional silver nanowires and two-dimensional MXene nanosheets has a very high sensitivity, with a GF factor of 220 at 1% strain, which was two orders of magnitude higher than that of similar devices. The supercapacitor is used as a ‘stealth’ power source to power the integrated E-skin system. Experimental results showed excellent sensing performance under dynamic and static deformation, regardless of the strain range. Once charged, the all-in-one E-skin can be applied to the human skin to detect real-time multi-scale human activities, including weak physiological signals such as pulse, swallowing, limb movements and a large range of limb motion as well. These bioinspired skins have a probable wide range of applications in rehabilitation equipment such as robotic rehabilitation training aids, artificial prosthesis-assisted functional rehabilitation and bioactive material-assisted functional rehabilitation to detect patient physiology at the same time they may assist healthcare professionals in various rehabilitation treatment activities (Yang et al., 2022). Professor Liu of Nanjing normal University (Shao et al., 2022) proposed a novel, conductive structure-colored composite hydrogel, which can be used to rehabilitate the skin of robot knuckles. The structure-colored composite hydrogel material has obvious color change and electromechanical properties in the bending process. Therefore, the film can be used as a multi-signal response of electronic skin to achieve real-time color sensing and electrical response, as well as for human finger joint rehabilitation robot. It is very valuable for many practical biomedical rehabilitation exercises. Coincidentally, Professor Zhao of Southeast University (Zhang et al., 2020), proposed a multi-functional E-skin inspired by chameleon. The E-skin uses a composite conductive cellulose liquid crystal hydrogel which can provide quantitative feedback for multiple stimuli through electrical signals and report the location of the stimulation site directly through color changes. This kind of dual-signal sensor offers visible interaction with users and anti-interference capacities, making it a prospective candidate for multi-function E-skin application with potential research value in healthcare and variable equipment. These bioinspired skins have a probable wide range of applications in rehabilitation equipment, including robotic rehabilitation training aids, artificial prosthesis-assisted functional rehabilitation and bioactive material-assisted functional rehabilitation to detect patient physiology while assisting healthcare professionals in various rehabilitation treatment activities (Yang et al., 2022).

### 4.3 Application of bioinspired skin in skin implants

**FIGURE 4:**
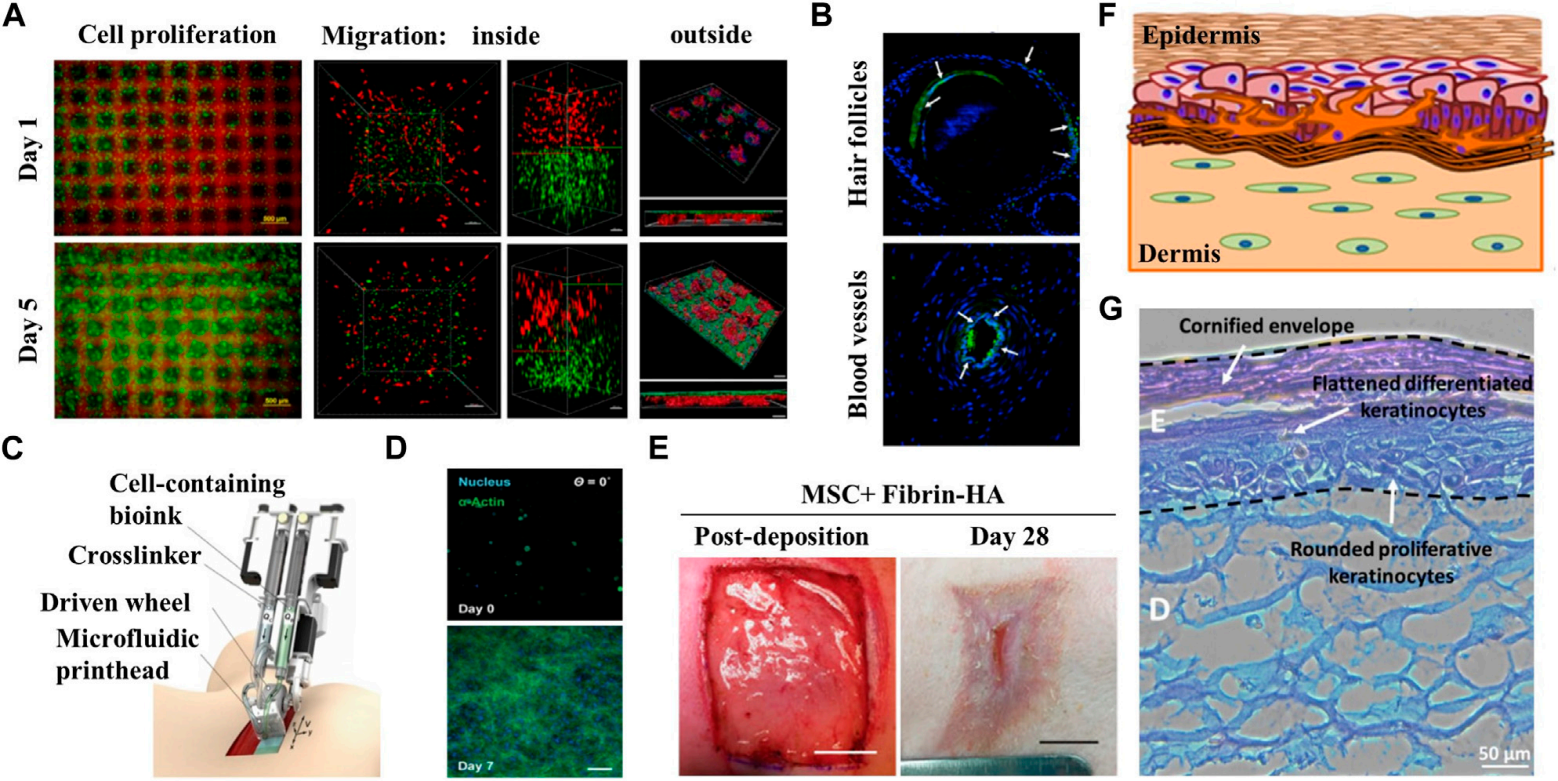
Application of bionic skin in burns/implants. **(A)** Microscope images of cell growth in the structure sustainable fabricated functional living skin (FLS) and analysis of cell migration and adhesion inside and outside of the bioprinting skin at day 1 and day 5. **(B)** Immunofluorescence staining showing the formation of hair follicles and blood vessels in FLS. Reproduced with permission from (Zhou et al., 2020). **(C)** Diagram of handheld instrument for controllable delivery of bionic. Reproduced with authorization from (Cheng et al., 2020). **(D)** 3D culture of MSC-fibrin-HA biomaterials at day 0 and day 7 after deposition showing the growth of MSC in biomaterial sheet. **(E)** *In-situ* deposition of MSC-containing fibrin-HA biomaterials into porcine full-thickness burn surface using the handheld device and recovery of the wound after 28 days. **(F)** Presentation of fabricated manual-cast 3D pigmented human skin constructs by bioprinting strategy. **(G)** H&E staining of pigmented human skin constructs obtained using the 3D bioprinting approach. Reproduced with permission from (Ng et al., 2018).

The primary application of bioinspired skin in skin implants is to repair damaged skin resulting from burns, wounds and scars to help to restore the function and appearance of the patients skin and improve their quality of life (Dearman et al., 2021; George et al., 2022). The group led by professor Zhang and professor Ouyang from Zhejiang University (Zhou et al., 2020) printed bioinspired skin with a unique structure by using a newly designed GelMA/HA-NB/LAP bio-ink and light-curing 3D printing technology. The interpenetrating pores within the bioinspired skin facilitate nutrient entry and oxygen exchange, which in turn promote cell adhesion, migration and proliferation leading to formation of new tissue (Figure 4A). *In vivo* studies have demonstrated the effectiveness of tissue-engineered artificial skin in promoting wound healing in both small and large animals, and superior regenerative performance for skin appendages such as hair follicles (Figure 4B). A team at the University of Toronto (Cheng et al., 2020) has developed a biological 3D printer which is a 3D printing device capable of generating large quantities of transplantable artificial skin in a relatively short time (Figure 4C). The printer allows for the extrusion of a hydrogel material known as a “living bandage”, which is a mixture of biopolymers, human keratinocytes (a type of skin cell) and fibroblasts. These cellular structures play principal role in wound healing. During the extrusion process, the hydrogel material is printed as discrete and well-defined honeycomb structures that mimic human skin. The application of this artificial skin tissue is aimed to cover the skin wounds caused by burns to promote skin repair, which could potentially revolutionize the rehabilitation process for burn patients (Figures 4D, E). On the other hand, scientists from the Singapore Institute of Manufacturing Technology (SIMTech) and the Centre for 3D Printing at Nanyang Technological University (SC3DP) have developed a 3D-printed fabricated colored skin with uniform skin pigmentation by using bio-3D printing technology (Ng et al., 2018). This is a significant advancement for the technology used in artificial skin manufacturing, as this technology is able to create artificial skin with a matching skin tone to the human body, which will facilitate the use of artificial skin technology in rehabilitation medicine and aid to increase confidence and self-esteem of the implanted patients (Figures 4F, G). Artificial tissue-engineered skin can play a crucial role in case of emergency by preventing wound infection and excessive dehydration of patients. In addition, the use of highly transparent artificial skin in follow-up treatment can also facilitate observation of effusion and infection (Aoki et al., 2015). Scar healing inevitably occurs in the process of skin healing. However, excessive scar growth may lead to scar hyperplasia, contracture, and even deformity, which can severely affect the appearance and function, and at the same time cause serious heart trauma. This can result in several problems in various aspects such as life management, social communication and daily work, and the use of artificial skin also plays a certain role in reducing scar. It is particularly important for the comprehensive rehabilitation of patients (Finnerty et al., 2016).

## 5 Discussion

The current mainstream works on electronic skin focus on enhancing a single or a few stimulus perceptions attributes, such as pressure or temperature and investigating the combination of some physicochemical and perceptual attributes. However, this type of work usually covers only two or three properties of the skin, which is still far from achieving a skin-like richness of sensory and physicochemical properties of stimulus perception. Hence, the next milestone towards more complex multifunctional bioinspired applications and the next-generation of artificial skin is to cover all the physicochemical and sensory properties of human skin (Duan et al., 2023). Although 3D-printed skin structures that exactly replicate natural skin have not yet been achieved, the current state of skin bioprinting already showed promises for creating functional skin equivalents in several key aspects, from the pre-processing stage to the evaluation of the final product (Yan et al., 2018).

At the same time, however, there are still several problems associated with bioinspired skin that need to be addressed. These include:

(1) Bioinspired skin has poor compatibility with human tissue structures due to the rich and diverse microstructure and complex physical and mechanical properties of human skin (Dąbrowska et al., 2016).
(2) The limited number of sensors loaded in the bioinspired skin results in its inability to sense signals powerfully and accurately from the surrounding environment, and further improvement and refine the ability of bioinspired skin for sensing the signal is depend on the continuous development of new technologies and materials (Du et al., 2020).
(3) At present, the bioinspired skin can only control mechanical movements, and there are still many challenges in the neural interface and brain-machine interface, making it difficult to achieve natural coordination between robots and the human body (Chortos et al., 2016).
(4) Current printed skin constructs are still distant from natural skin. In addition, addressing other ethical, regulatory and social barriers associated with bioprinting is essential (Tarassoli et al., 2018).

## 6 Conclusion and future perspective

Bioinspired skin has been used in a wide range of applications in the field of rehabilitation medicine, including prostheses, rehabilitation and burns/implants (Wang et al., 2021; Peng et al., 2023). The field of rehabilitation medicine has benefited most from the application of electronic skin particularly in two key aspects. Firstly, as an external control system, the electronic skin is precisely designed with high flexibility and low friction coefficient, so that it can be used as an external drive system for medical robots, through its control signal to achieve the precise operation of the robot (Yu et al., 2021). Secondly, sensors or micro control systems can be utilized to improve human-robot interaction (Zhang M et al., 2022). In the case of rehabilitation robots and prostheses, haptic sensors would allow rehabilitation robots or amputees to detect their physical surroundings, enabling the performer to handle and manipulate everyday objects and interact with other people among other tasks (Wang et al., 2023).

In the future, bioinspired artificial skin will have the ability to learn and perceive its environment, similar to human skin. It will be able to sense its surroundings and provide feedback to the brain through various sensing signals. For instance, when it comes into contact with other objects in the environment, it will sense the presence of that object and provide feedback to the brain accordingly, so that it can exist like natural skin, and even surpass its capabilities. To this end, the next step in the research should integrate computer intelligence technology for the design and production of artificial bioinspired skin, broaden the application of bioinspired skin, and bring new prospects in the field of rehabilitation medicine (Zhang et al., 2019; Zhang *p* et al., 2021). At the same time, researches should be extended with complex artificial skin having multifunctional physicochemical and sensory perceptions similar to human skin. The close collaboration of multidisciplinary and high-precision technologies will eventually make the extensive application of bioinspired skin in rehabilitation medicine a reality.
