# Supplementary figures and images for: Bioinspired skin towards next-generation rehabilitation medicine
Source: Front Bioeng Biotechnol. 2023 May 9;11:1196174. doi: 10.3389/fbioe.2023.1196174 (PMC10203386; doi:10.3389/fbioe.2023.1196174)

## Slide 1
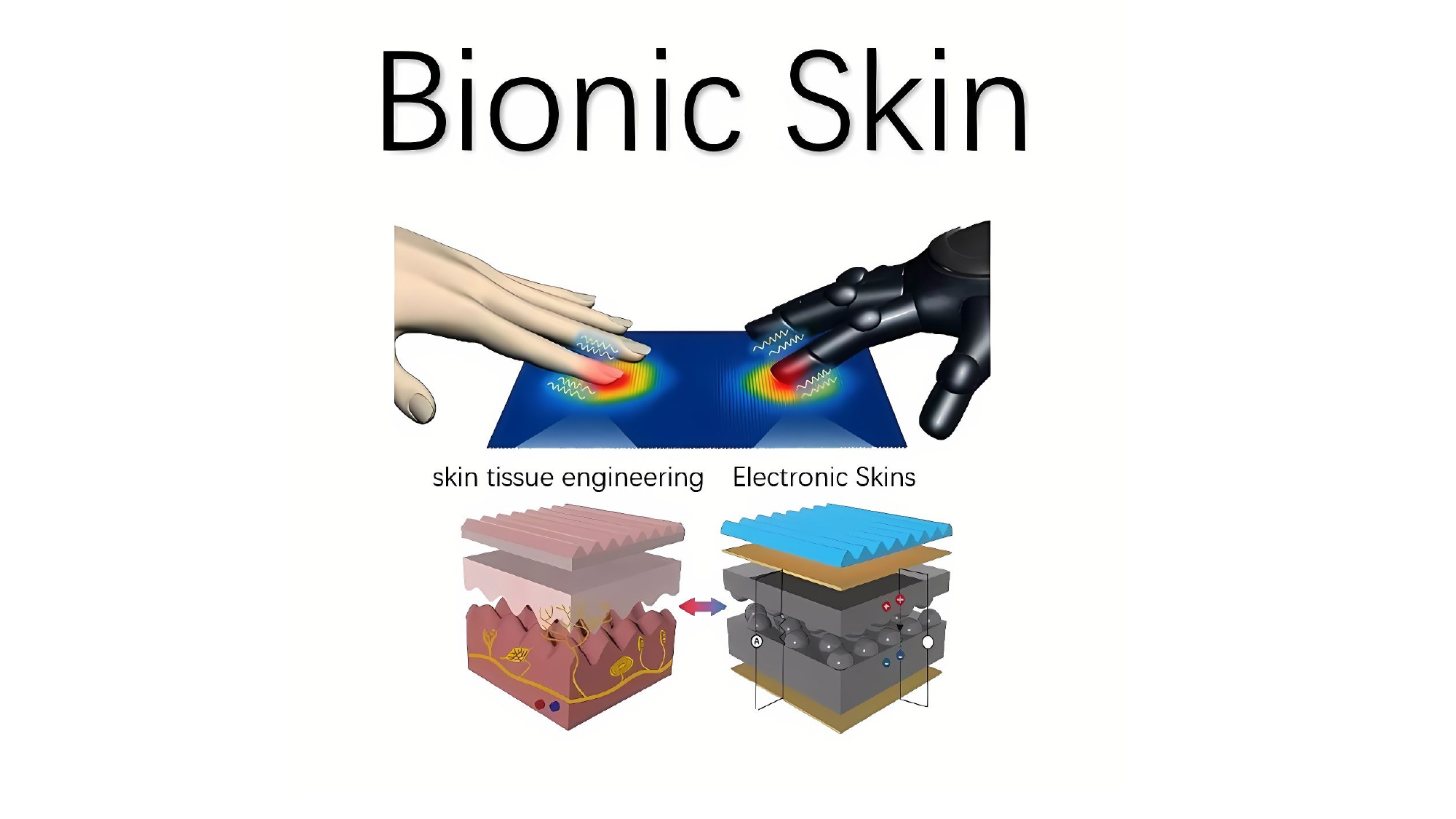

## Slide 2
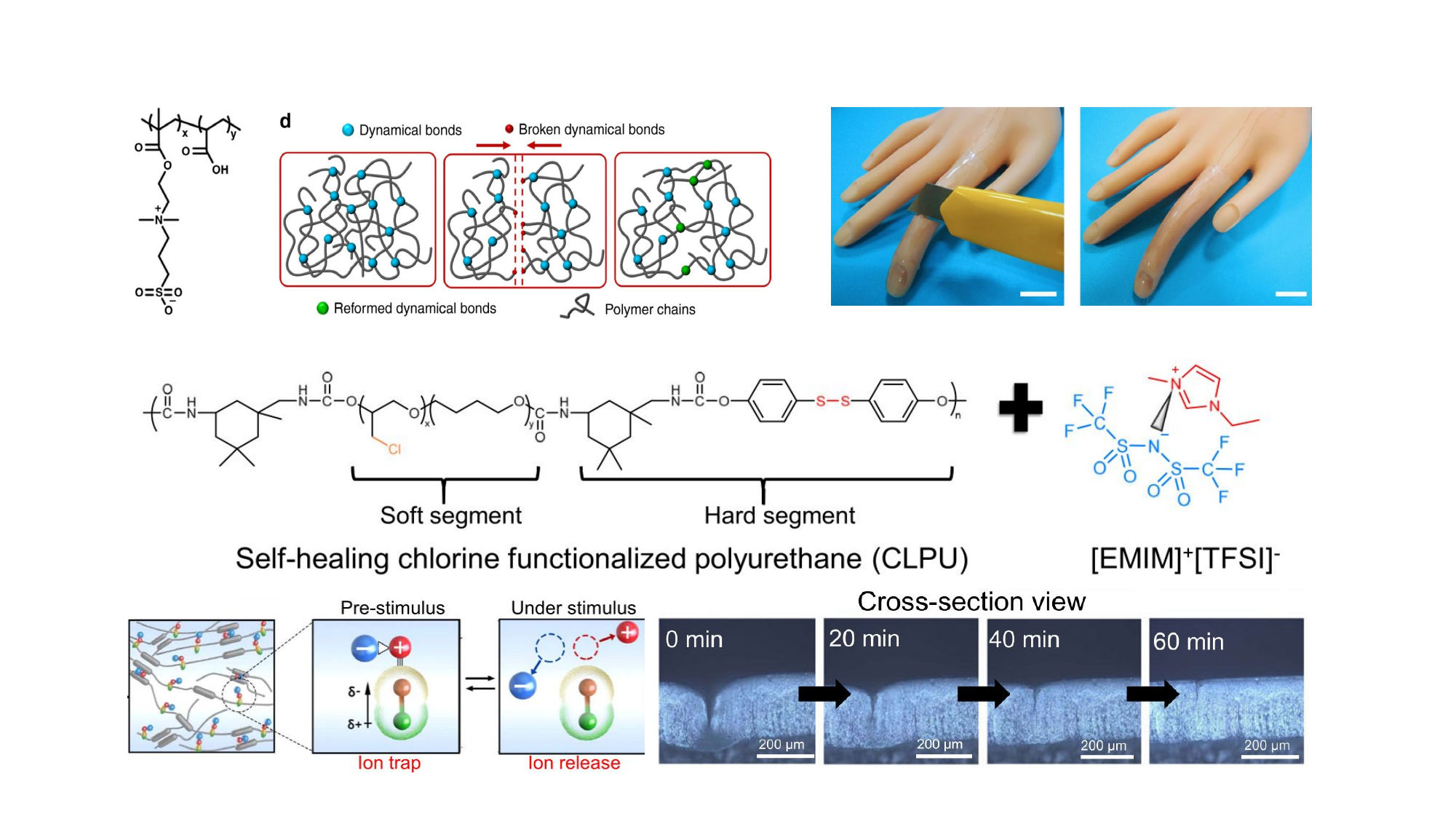

## Slide 3
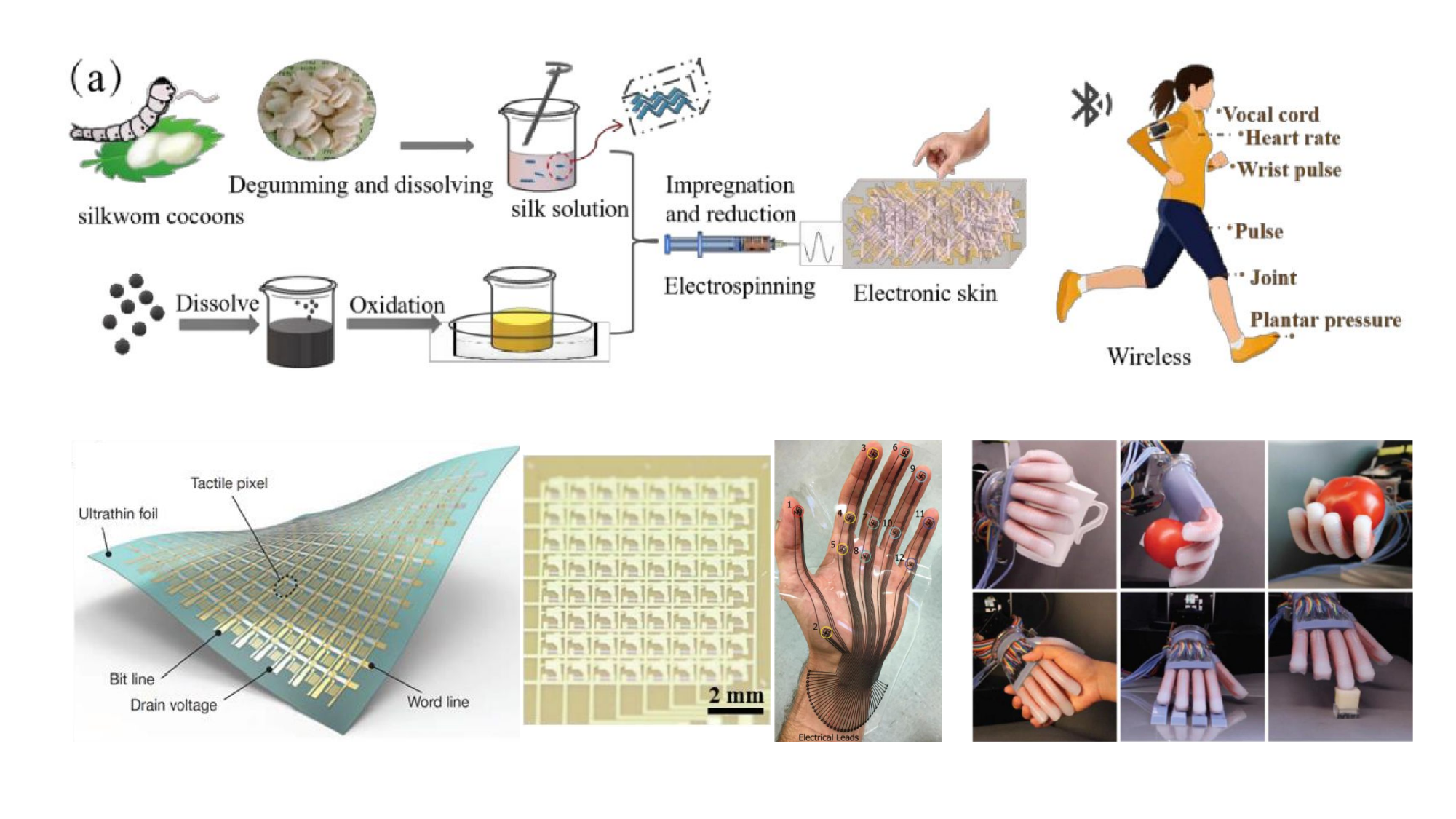

## Slide 4
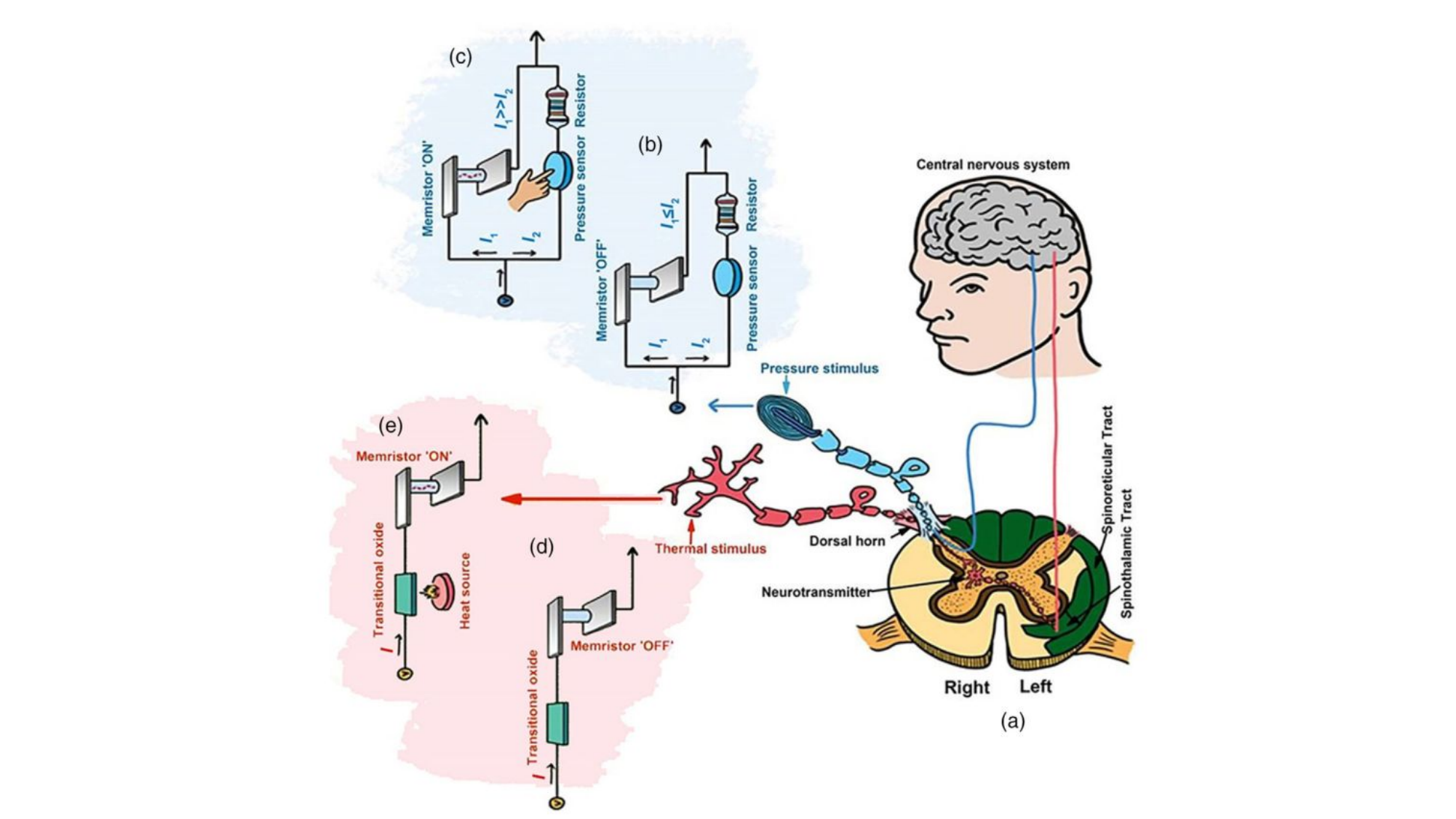

## Slide 5
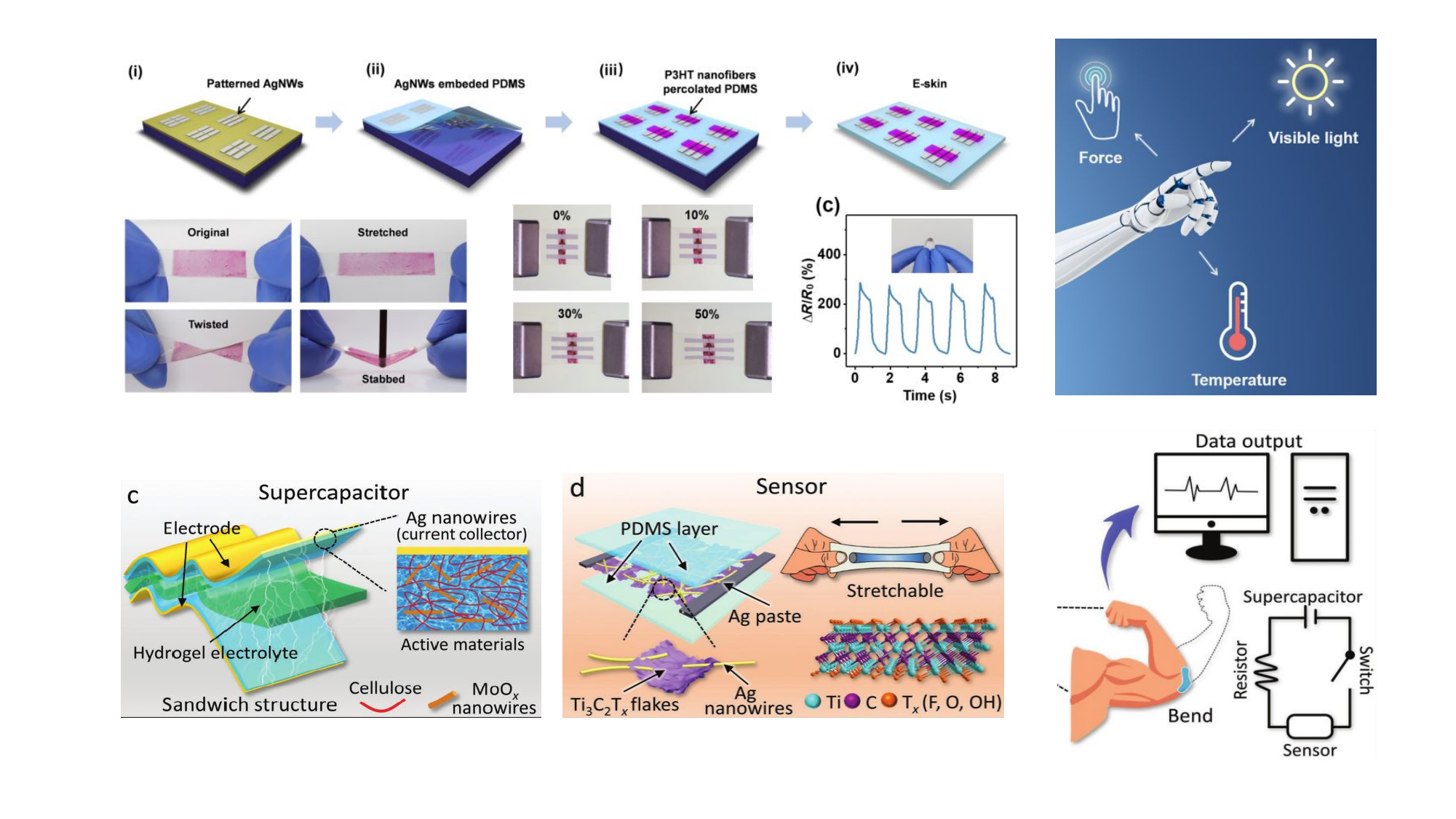

## Slide 6
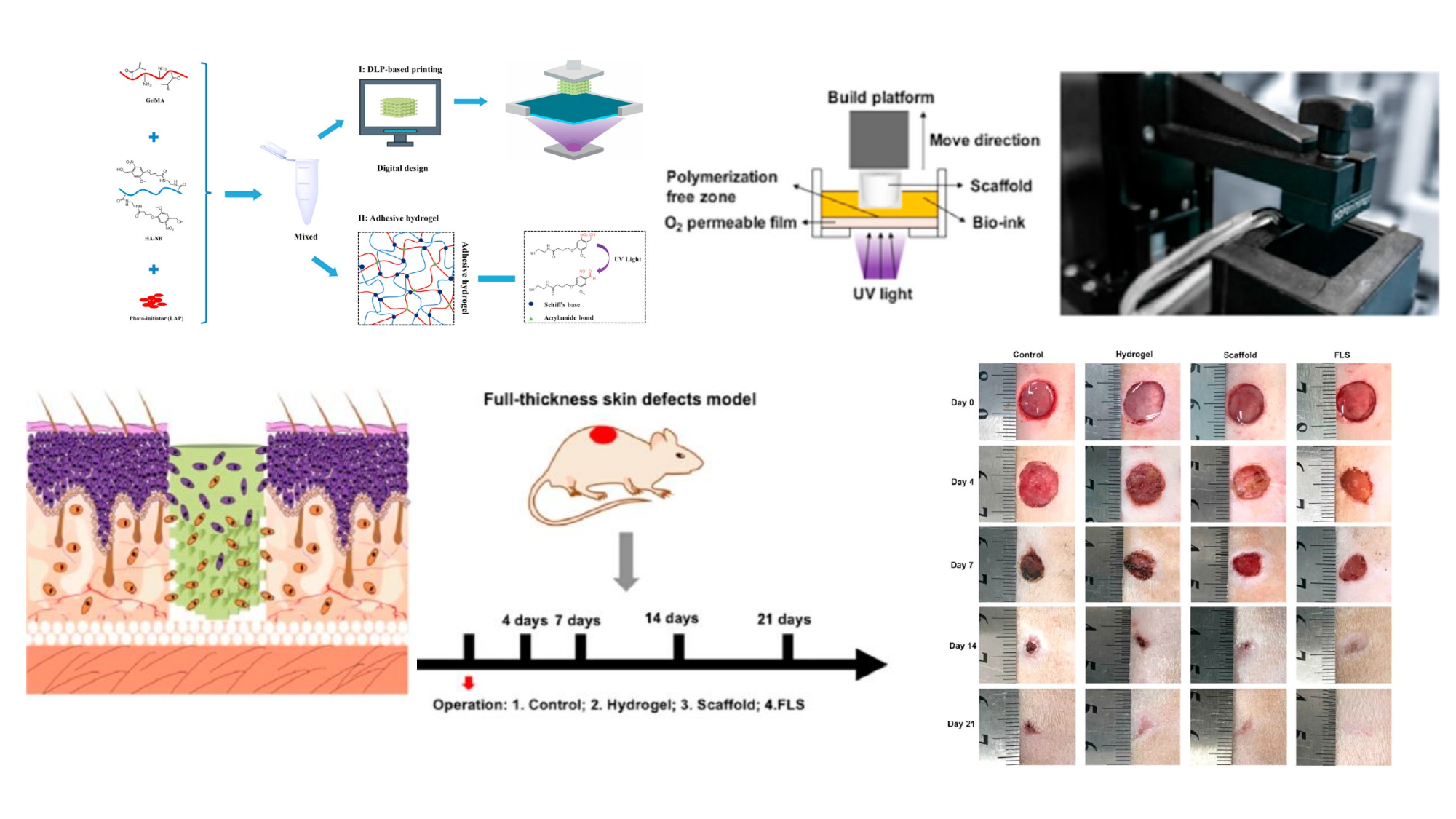

## Slide 7
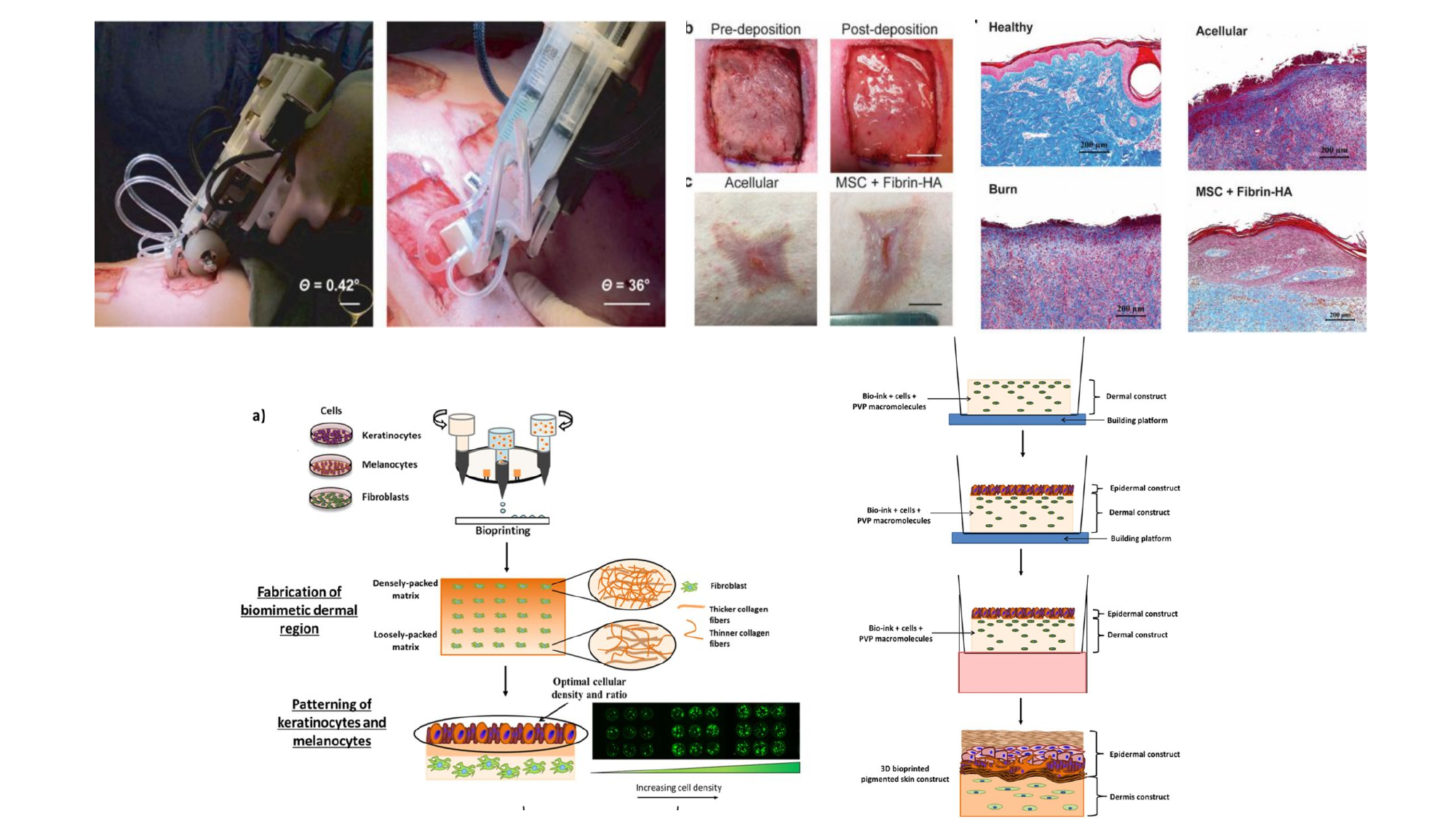

## Slide 8
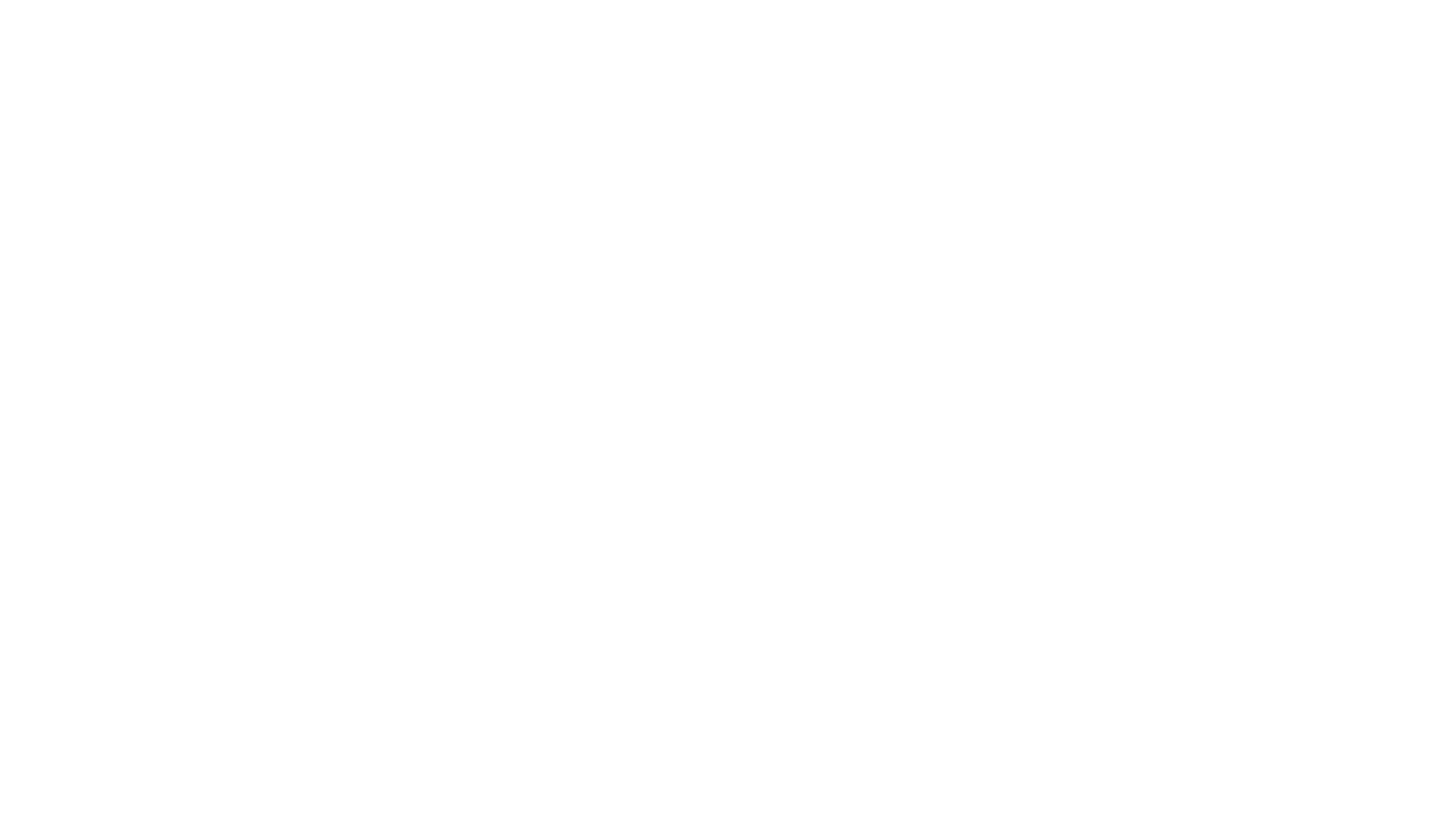

Supplement: Supplementary file 1 [file Presentation1.PPTX]
